# Retraction

**DOI:** 10.1111/cas.15808

**Published:** 2023-04-25

## Abstract

Retraction: Le Kang, Jun Mao, Yajun Tao, Bo Song, Wei Ma, Ying Lu, Lijing Zhao, Jiazhi Li, Baoxue Yang, Lianhong Li, MicroRNA‐34a suppresses the breast cancer stem cell‐like characteristics by downregulating Notch1 pathway, Cancer Science 2015, 106 (6), pp. 700–708 (https://onlinelibrary.wiley.com/doi/10.1111/cas.12656).

The above article, published online on 17 March 2015 in Wiley Online Library (wileyonlinelibrary.com) has been retracted by agreement between the authors, the journal Editor in Chief Masanori Hatakeyama, Japanese Cancer Association and John Wiley and Sons Australia, Ltd. following an investigation on overlapping images in Figure 3B. The authors asked to retract this manuscript as the experimental data in the article could not be reproduced as the original data are no longer available. Therefore, the article's conclusions cannot be verified and must be considered unreliable.

Retraction: Le Kang, Jun Mao, Yajun Tao, Bo Song, Wei Ma, Ying Lu, Lijing Zhao, Jiazhi Li, Baoxue Yang, Lianhong Li, MicroRNA‐34a suppresses the breast cancer stem cell‐like characteristics by downregulating Notch1 pathway, Cancer Science 2015, 106 (6), pp. 700–708 (https://onlinelibrary.wiley.com/doi/10.1111/cas.12656).

The above article, published online on 17 March 2015 in Wiley Online Library (wileyonlinelibrary.com) has been retracted by agreement between the authors, the journal Editor in Chief Masanori Hatakeyama, Japanese Cancer Association and John Wiley and Sons Australia, Ltd. following an investigation on overlapping images in Figure 3B. The authors asked to retract this manuscript as the experimental data in the article could not be reproduced as the original data are no longer available. Therefore, the article's conclusions cannot be verified and must be considered unreliable.

